# The Sequential Binge, a New Therapeutic Approach for Binge Eating: A Pilot Study

**DOI:** 10.1371/journal.pone.0165696

**Published:** 2016-11-10

**Authors:** Rémi Neveu, Dorine Neveu, Guillaume Barbalat, Ulrike Schmidt, Giorgio Coricelli, Alain Nicolas

**Affiliations:** 1 CNRS, UMR5292, INSERM, U1028, Université Lyon 1, Université de Lyon, Neuroscience Research Center, Team Olfaction: from coding to memory, Lyon, F-69366, France; 2 Praxis, Ville-la-Grand, France; 3 Université Montpellier 1, Montpellier, France; 4 INSERM U 1058, Montpellier, France; 5 CHU Montpellier, Département d'information médicale, Montpellier, France; 6 Regional Eating Disorders Service, Greenlane Clinical Centre, Auckland, New Zealand; 7 Section of Eating Disorders (PO59), Institute of Psychiatry, King's College London, De Crespigny Park, London, SE5 8AF, United Kingdom; 8 University of Southern California, Los Angeles, CA, United States of America; 9 Interdepartmental Centre for Mind/Brain Sciences (CIMeC), University of Trento, Trento, Italy; 10 Hôpital du Vinatier, Bron, France; Hospital Universitari de Bellvitge, SPAIN

## Abstract

**Background and Objectives:**

A sizeable proportion of patients experiencing binge eating do not respond to cognitive behavioral therapy (CBT). We present the sequential binge (SB), a new behavioral intervention that complements CBT, and preliminary results of its effects. SB breaks up the binge into repeated identical sequences of eating separated by incremental pauses. This pattern of ingestion aims at facilitating boredom toward the ingested foods and at turning cognitive control away from binge food restriction. SB is hypothesized to reduce food intake during the binge and the number of daily binges.

**Methods:**

Prospective pilot study. Fifteen binging patients with previous unsuccessful intensive CBT were given SB as an adjunct to their treatment and were followed up for 16 weeks from admission. All patients were reassessed 47 weeks on average after discharge.

**Results:**

SB was associated with a 44% relative reduction in the planned food intake (p<0.001), a longer consecutive binge refractory period compared to regular binges (median: 48 hours versus 4 hours, p = 0.002) and an average relative reduction by 26% of binge number the day after each SB (p = 0.004). 47% of patients reached binge abstinence for four consecutive weeks 16 weeks after the first SB.

**Conclusion:**

This case series shows promising evidence for the use of SB in patients with refractory binge eating. Further evaluation in a prospective randomized controlled trial would be justified.

## Introduction

Binge eating is defined as “recurrent periods of uncontrolled overeating” [1]. It causes significant distress in patients with bulimia nervosa (BN), anorexia nervosa binging subtype (ANB), binge eating disorder (BED) or Other Specified Feeding or Eating Disorder [1]. According to operant conditioning theory, the occurrence of binges is mainly triggered by strict dieting between binges, which results from patients’ over concern about weight, shape and their control [1]. In turn, binge eating further reinforces strict dietary rules as well as overvaluation of weight, shape and their control. Cognitive behavioral therapies (CBT) address this deleterious circle by suppressing the reinforcing processes of binge occurrence: strict diet and over concern about food, weight and shape [1].

However, only 40% to 50% of BN and BED patients recover from binge eating at the end of a CBT course [2,3] and interventions recently developed do not improve much binge frequency when they complement CBT [4,5]. This partial efficacy could stem from the fact that CBT and these interventions neglect the processes occurring specifically *during* binges. These processes facilitate a subsequent restriction of binge foods and reinforce binge occurrence [1]. These processes are i) the excessive food intake, which temporarily reduces the desire to eat these foods through a saturation effect [6–9]; ii) the use of planning and other cognitive control abilities during the binge to reduce long-term binge food attractiveness [10,11]; and iii) the overall feeling of loss of control at the end of the binge which is consecutive to the excessive food intake, feeling that strengthens subsequent binge food restriction [1].

Here, we present a new behavioral intervention, the sequential binge (SB), that would tackle the three aforementioned processes, and could potentially benefit patients who did not improve with CBT. As recommended when developing new complex treatments [12], this study aims at describing SB and its theoretical underlying mechanisms, testing its feasibility with a pilot group of binging patients and providing a proof of concept with preliminary results.

The SB intervention consists in replacing patients’ usual pattern of food ingestion during a binge by a repeated monotonous food ingestion sequence, interspersed with short incremental pauses. The sequence is set at every new binge without constraints on food choice or compensatory behaviors. Compared to a regular binge, only the sequence of food ingestion is changed. Such an intervention may have an effect on the three processes described above. First, the monotonous repeated ingestion of a sequence of foods aims at promoting boredom toward the foods ingested during the binge and thus at reducing food intake [8,9]. Second, the rules of ingestion during SB aim at training patients to keep focused on a goal that is unrelated to binge food restriction. Specifically, patients would have to make their food rules more flexible, reorient planning abilities and ignore task irrelevant information [10,13–15]. Pauses aim at training patients on command to inhibit the dominant compulsive ingestion of binge foods [10,16]. The increment in pause duration aims at training patients to promote a later intake of reasonable quantities of binge foods when facing the desire to eat these foods (instead of an immediate ingestion of excessive quantities) [15,17]. Without this increment, pauses might be automated and only promote the inhibition of the rash food intake [17]. Overall, the reorientation of cognitive control skills and the reduction of binge food intake should reduce the feeling of loss of control patients experience at the end of the binge.

Through its rules of ingestion, SB resembles a meal-like structured ingestion of binge foods [18,19] but differs fundamentally in its goal. A meal-like structured ingestion of binge foods aims at promoting positive feelings during binge food ingestion. SB however aims at reducing food intake and reorienting cognitive control skills during the binge without looking for experiencing the binge as a pleasurable meal [18,19]. SB also differs from CBT and mindful eating. Indeed, SB does not promote an alternative behavior to the symptom as CBT would [19]. SB also sets an active behavioral response to the binge impulse whereas mindfulness promotes a passive observation of events [20].

SB aims and mechanisms let us hypothesize that SB would reduce binge food intake during binges, daily binge number the day after each implementation and increase the binge refractory period (the period without any binge craving) after each SB in a pilot group of ANB, BN and BED patients.

## Methods

### Population

Between September 2008 and October 2010, in-patients or day-patients were recruited from two specialist eating disorders units (Clinique des Vallées and Clinique Belmont). Patients were eligible in the study if 1) they had an eating disorder diagnosis where binge eating was prominent from the DSM-IV (i.e. BN, ANB, BED or EDNOS with significant binge eating) [21]; and 2) they had been unresponsive to CBT. First inclusion criterion was ascertained at patients’ admission by a psychiatrist through a structured interview. The second inclusion criterion was fulfilled if i) patients experienced a persistent strong distress related to binges that demotivated them and would have jeopardized the pursuit of CBT; or ii) they demonstrated a relative reduction of binge frequency of less than 50% after at least four weeks of intensive CBT within the unit [22]. There was no exclusion criterion.

**Fig 1:**
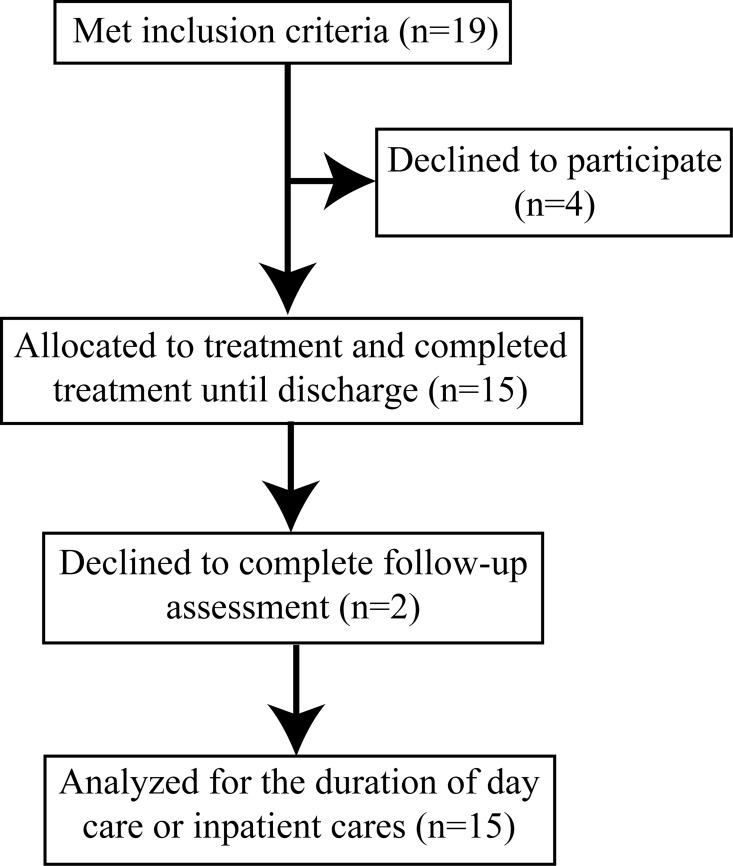
Flow chart of inclusion of patients.

Nineteen patients were eligible for the study. They were individually given oral and written information by the therapist about the study and the SB intervention. Of those 19 patients, 15 were interested in implementing SB (1 day-patient, 14 in-patients; 6 ANB, 8 BN and 1 BED, Fig 1), which led to an acceptance rate of 79%. All enrolled patients gave their signed informed consent before enrollment. They were introduced to SB after a median duration of 27 days after admission (interquartile: 20–56). The poor reduction in binge frequency (i.e. number of binges over the past 4 weeks) between admission and the end of the first four weeks of intensive CBT confirmed the second inclusion criterion (mean reduction (range): 10% (-6% to 27%)). A high binge frequency over the past 28 days characterized enrolled patients when being introduced to SB (median: 42, interquartile, 30–71). The majority of participants (n = 9) had psychiatric comorbidities associated with a poor prognosis of remission or recovery such as substance–related, major depressive, bipolar or borderline personality disorders (see Table 1 for more information) [23–26].

**Table 1:** Participants’ socio-demographic and clinical characteristics. Medians (interquartile) are reported, otherwise specified.

	Pilot group (n = 15)
Age at SB presentation,years	24 (22–27)
Female gender, n (%)	14 (93)
Education, years	13 (12–14)
Brothers, n	1 (0–2)
Sisters, n	1 (0–2)
Eating disorder diagnosis	
- Anorexia Nervosa	6 (40%)
- Bulimia Nervosa	8 (53%)
- Binge eating disorder	1 (7%)
Psychiatric co-morbidities	
- At least one, n (%)*	12 (80)
- Associated with poor prognosis, n (%)**	9 (60%)
History of anorexia nervosa, n (%)	11 (73%)
BMI at first SB use, kg/m^2^	18.2 (16.6–20.4)
Time from disorder occurrence to SB presentation, years	11 (6–13)
Time from disorder diagnosis to SB presentation, years	6.1 (2.9–9.8)
Number of binges over the past 28 days at SB presentation	42 (30–71)
Number of binges over the past 28 days at first SB use	32 (27–64)

Abbreviations: SB: sequential binge; BMI: body mass index.

*: substance related disorder, social phobia, post-traumatic stress disorder, obsessive compulsive disorder, parasomnia due to restless leg syndrome, major depressive disorder, bipolar disorder, dependent personality disorder, borderline personality disorder.

**: substance related disorder, major depressive disorder, bipolar disorder, borderline personality disorder.

The study was approved by an independent research ethical committee (Comité de Protection des Personnes Sud Est IV).

### Protocol

#### Outlines

Patients were enrolled in a multiple baseline prospective study. The period between admission and the first SB was the baseline reference. Because patients could choose not to implement SB at every binge, the period ranging from first SB to discharge constituted an ABAB-like design, with A referring to days with at least one SB and B referring to days without any SB. Note that B days could refer to days without any binge or to days with regular binges only and are control periods (as is the baseline reference).

During their stay at the hospital that lasted for 16 weeks (median, interquartile: 12–34), patients received individual CBT as designed for inpatient settings [19]. Patients also engaged in a multi-faceted therapeutic group program designed to enhance their cognitive, behavioral, nutritional and emotional skills. BN or BED patients also received high dose SSRI (Selective Serotonin Reuptake Inhibitor) as recommended [2,27].

We chose an inpatient rather than outpatient setting for the following reasons. First, an inpatient setting allows fast reactivity of the medical team in case of poor compliance to the technique or major adverse events (two patients had a clinically significant depressive episode after the onset of SB) [28,29]. Second, a substantial proportion of our patients had AN, psychiatric co-morbidities, and/or a large number of binge episodes at admission. According to literature, those patients are more likely to fail to respond to CBT [23–26] and require a more intensive program in an inpatient setting [19,30].

Until the first SB, CBT was complemented by at least one binge management strategy. Binge management strategies were stopped just before the first SB to avoid any interaction with SB. However, CBT, SSRI and the multi-faceted group program were continued. The rationale for continuing CBT and SSRI was that SB does not target the core mechanisms occurring outside binges, such as dietary restraint and overvaluation of weight, shape and their control.

All patients were contacted for a reassessment 47 weeks ± 7 weeks after discharge.

#### CBT and binge management strategies

Since admission, all patients received individual sessions of one hour of CBT-E for eating disorders according to Fairburn [19]. They were delivered twice weekly by either a psychiatrist with 11 years experience, or a psychologist with 8 years experience. This was complemented by a French translation of Fairburn’s self-help book [18,31]. This book allowed patients to efficiently prepare the upcoming sessions of CBT-E. CBT-E included three main successive steps: first, therapists provided psycho-educational information about the reinforcing mechanisms of the disorder. In addition, patients were asked to monitor their daily food intake while they had three calibrated daily meals complemented by one or two snacks at set times. Secondly, excessive or dysfunctional body image, strict dieting and relationship between life events, mood and eating were identified and challenged with cognitive and behavioral exercises (e.g. thought challenging, behavioral exposures and problem solving). Third, relapse prevention strategies were developed [19]. Regular reviews were conducted throughout the treatment. Likewise the progress reviews, potential barriers to change and motivational issues were regularly discussed and addressed if necessary.

Step 1 of CBT-E included the use of distraction techniques when patients faced the urge to binge [19]. This was complemented with other binge management techniques tailored to the patients that included: bingeing with non-binge foods; ‘scheduled binges’ (patients binged only on days pre-arranged with the therapist); and/or nasogastric feeding for a minimum one month period [32].

The medical team did not exert any control over binges or regular eating. Patients could binge during their stay at the hospital as they had access to foods.

#### The sequential binge (SB)

The SB protocol consisted of 3 distinct steps that were explained to the patient. As the urge to binge is not synchronous among patients, the SB protocol was set individually.

#### Step 1: Packaging foods

Patients were asked to gather all the foods they had planned to eat during their binge, as they would have done for a regular binge. Then patients had to share the food out into 6 to 10 identical packages, each package including a portion of each binge food. Packages could be prepared either before the binge (e.g. several hours or days) or when the urge to binge occurred, depending on the patients' habits. All packages had to be identical regarding food types, brands and quantity of food, although the amounts of food were allowed to differ from one food type to another in the same package. As binge food craving transiently increases at the beginning of the binge [33], the amount of food in the first package had to be large enough so that the end of the ingestion of the first package occurred after this transient increase.

As women ingest on average 642 kcal during each meal and the total intake of a binge is 3469 kcal on average [7,34], we calculated that sharing out food into 6 to 10 packages corresponded to 50–90% of an average meal for each package. This fulfilled the objective of promoting a reasonable amount of food in one intake. As the duration of a regular binge is 41 minutes on average [7], the ingestion of each package should last between 4 and 7 minutes.

#### Step 2: The food ingestion sequence

**Fig 2:**
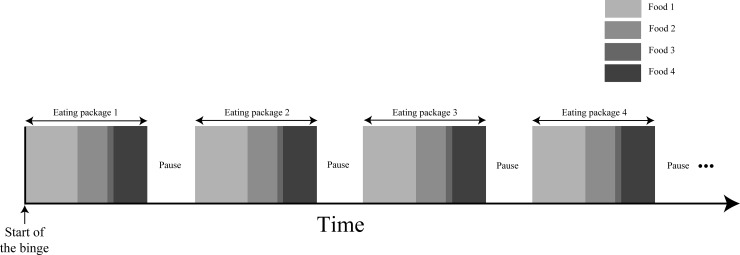
Time order of food ingestion during the sequential binge (example with 4 binge foods and 4 packages).

Food was ingested sequentially with incremental pauses between packages. The time order of food ingestion is presented in Fig 2.

**Ingestion:** All prepared packages were stored out of sight but could be easily accessed (for instance in a corner of the room where the binge takes place) except for the first package which was eaten without delay. Food in the first package was eaten without any predefined order. However, the sequence of ingestion for the first package had to be reproduced for the subsequent packages.

**Pause:** After the first package was eaten, patients had to pause. During this pause, they were allowed to engage in any activities provided they were not related to food, not a compensatory behavior, not a physical activity and intellectually not too demanding. Indeed, any compensatory behavior or physical activity during pauses might lead patients to experience the intake of each package as an independent binge and thus would strengthen the feeling of loss of control at the end of the binge. Setting an activity unrelated to food during pauses aimed at disrupting the automated ingestion of binge food which usually happens in a regular binge. Conversely, an activity that is too cognitively demanding would capture too much attention and thus would reduce the ability to recall the sequence. As a distractor, the concurrent activity, whatever its nature, was also expected to reduce craving for the selected food [35]. Each package had to be entirely finished before pausing and moving on to the next one.

The physical move required to get the next package also aimed at preventing an automated ingestion of food [36].

The duration of pauses was incremental for the first three packages at least, and stabilized for the remaining packages. Pauses were incremental at the beginning of SB to prevent their automation during SB (which would then resemble a regular binge interspersed with regular pauses). However, if pause duration was to increase till the end of SB, patients would learn to permanently delay food ingestion, which in turn could promote food restriction. Instead, stabilizing pause duration towards the end of SB would promote delayed ingestion (rather than restriction) of a reasonable amount of food [17].

Package size and pause duration were constrained by the time course of the specific desire to eat one food. The desire to eat a specific food progressively decreases during its ingestion [37]. Then, the desire slowly increases back to its initial value about an hour after the ingestion has stopped [37].

Over SB, a gradual decrease of the cumulative desire to eat a specific food could only occur if, for each package, the food intake lasted substantially less than the duration of this craving reset. If the desire to eat the selected food is reset to its initial peak, boredom could never occur. Overall, pauses were not too long to satisfy this constraint i.e. optimally elicit the occurrence of boredom for binge food (typically 120 seconds, lower than 300 seconds). Note that the first pause was chosen to last 10 seconds as patients might not be able to stand longer pauses (craving for binge food is still intense at the end of the first package).

#### Step 3: End of the binge

Binge ended at the patients' convenience, i.e. at any time during SB. Spared binge food was either disposed of or stored for a subsequent binge (regular or SB) at the patients' convenience.

At the end of each SB, a therapist reviewed that the process was adequately implemented in a face-to-face interview with the patient. Of particular importance were i) the number of packages stored out of hand and the sequence of ingestion from one package to another; ii) pauses between two packages: duration, activities during pauses, compensatory behaviors, physical activities; iii) potential food skipped; iv) variation of the sequence of ingestion across SBs; v) rule sets for choosing foods for SBs that would have differed from a regular binge.

Patients were encouraged to implement SB whenever they had an urge to binge. Encouragement was reiterated after two consecutive regular binges.

To prevent any automation of food ingestion during SB, the parameters of SB (pause duration, types of food and sequence of ingestion) were changed before each implementation.

Note that the medical team was allowed to help patients during the first step only.

### Assessment

Binges were defined as any food ingestion perceived as large and experienced with a feeling of loss of control. This definition refers to subjective binge eating [38]. Compared to objective binge eating which is based on the objective amount of food ingested, subjective binge eating better reflects the core of patients’ distress [38]. With its design, SB can be implemented only if the patient intends to binge with a large amount of foods. However, SB is hypothesized to impact subsequent episodes of food intake with a feeling of loss of control whatever the amount of food and perceived by the patient as a binge. Therefore we collected subjective binges that include all these episodes.

Information about binge characteristics (i.e. SB vs. regular binge), compensatory behaviors, medical treatment and patients' feedback were collected routinely by nurses specialized in the treatment of eating disorders. Patients daily self-monitored the type and amount of foods they ingested, the time and place where they started and finished eating, the nature of the binge (regular vs. sequential), any purging behaviors and their feelings and comments [19]. In an additional standardized self-administered questionnaire built for the purpose of the study, patients reported the binge refractory period (for both SB and regular binges) and the number of packages prepared and ingested at each SB. The amount of food planned for each SB was a reliable measure of the food intake during binges because almost all patients usually eat the amount of food they have planned [39,40].

Patients’ notebooks and information collected by the medical team were used to compute daily binge number. If those differed, only information on patients’ notebooks was used. Note that assessing binge number with standardized questionnaires such as the Eating Disorder Examination Questionnaire [19] would not be suitable for our study. Indeed, these questionnaires collect the total number of binges over a period of 28 consecutive days which is not appropriate for investigating immediate effects of our intervention on daily binge number.

Height was measured once at admission and weight was measured weekly. Socio-demographic data at enrollment were collected in a standardized self-administered questionnaire.

During the follow-up assessment occurring 47 weeks on average after discharge, the current clinical status and the disorder evolution and treatments since discharge were collected through a structured clinical interview.

### Statistical analysis

All enrolled patients were included in the present analysis. Analysis was carried out in four steps.

#### Step 1

We investigated the characteristics of implementation of the first SB: the time between SB presentation and first SB; binge frequency at SB presentation; reduction in binge frequency between admission and SB presentation; and their associations. Associations were appraised with the Spearman correlation coefficient. This step of the analysis also aimed at confirming the ABAB like design. Specifically, we tested whether the number of binges the day before each SB was significantly different to the number of binges the day before the first SB. We ran a linear mixed model in which the number of binges the day before SB was modeled as a function of SB index (“first” for first SB vs. “following” for all the other SBs). Participants were modeled as a random factor(model 1).

#### Step 2

We investigated the immediate effects of SB on the quantity of binge food ingested, the binge refractory period and the daily binge number. The relative reduction of food intake was estimated as the ratio of the number of packages remaining at the end of SB to the initial number of packages. The Wilcoxon test was used to compare the duration of the refractory period after SBs vs. regular binges. The association between the reduction in food ingestion and the relative binge refractory period (computed as the ratio of the individual average duration of the binge refractory period after SB to the one after a regular binge) was assessed with the Spearman correlation coefficient.

We then analyzed the effect of SB on daily binge number. The period from admission to the first implementation of SB (exclusive) was reported as the pre-SB period. The period from the first implementation of SB to discharge was reported as the post-SB period. The periods during which SB was not implemented (i.e. the pre-SB period and days without SB over the post-SB period) were control conditions for each participant. We labeled each day with SB as “SB day” and each day SB was not implemented as “no-SB day”. There was a series of “SB days” and “no-SB days” for each patient.

The immediate effect of SB on the day-to-day relative variation in daily binge number was assessed with a linear mixed model (model 2) as recommended for longitudinal data [41,42]. This model included daily number of binges as the dependent variable and the following independent variables: (1) number of binges the previous day, (2) use of SB (“SB day” vs. “no-SB day” during the pre-SB period vs. “no-SB day” in the post-SB period), (3) interaction between (1) and (2), (4) age at first diagnosis [26,43], (5) time elapsed between first diagnosis and SB presentation [43], (6) diagnosis (AN versus BN and BED together) [44], (7) co-morbidities associated with impulsivity or mood [23] (presence/absence of substance–related, major depressive, bipolar or borderline personality disorder), and (8) educational level [24]. Participants were set as a random factor over the intercept. Note that only data ranging from 10 days before first SB to 38 days after first SB were included in the model. This cutoff was chosen in order to elicit the same period of follow-up in all participants.

As each patient implemented SB several times and CBT every day, this model allowed us to extract the specific contribution of SB to the reduction in daily binge number after its implementation compared to CBT, and whether these variations were associated to the number of binges the day before.

We then investigated whether SB effect on the reduction in daily binge number was entirely or not entirely mediated by the reduction of binge food intake during SB. A regular mixed model is not appropriate to assess this effect [45]. We used the sequential g-estimator approach developed by Vansteelandt applied to model 2 complemented with the reduction of food intake on days with SB [45](model 3).

For model 2 and model 3, missing data for daily binge number were linearly interpolated with the nearest available daily binge number. Daily binge number included both regular binges and SBs in this interpolation.

We also ran three other imputation methods to assess the robustness of the results. The first one was the same as the aforementioned interpolation method except that SBs were not included in the calculation of daily binge number (they were however added after the interpolation) (imputation A). The rationale behind this method is that SB might have been better recorded as they were the main focus of the study. The other methods included the replacement of all missing data by the closest available daily binge number before the missing data (imputation B) or following the missing data (imputation C).

To visualize the results obtained with the mixed models, we plotted the daily binge number as a function of the time interval elapsed between two consecutive SB. First, we computed the relative daily binge number of a day by dividing the daily binge number of that day by the daily binge number of the previous SB day. Second, time between two consecutive SBs (e.g. SB A and SB B) was transformed into percentages so that a time interval of 0% corresponds to SB A day and a time interval of 100% to SB B day. For each percent of time, a paired t-test was computed to compare each relative daily binge number to that of SB A day (i.e. 100% of daily binge number). Multiple comparisons were accounted for with Benjamini false discovery rate.

#### Step 3

This step investigated the long term outcome of patients. It encompassed potential side effects, recovery (defined as no binges over four consecutive weeks) and remissions (defined as less than two binges per week over four consecutive weeks). No further analysis was carried out in this step because the study design does not allow any inference about a causal relationship with SB and it was not the goal of the study.

#### Step 4

We analyzed any spontaneous feedback reported by the patients that was not assessed systematically by questionnaires or interviews.

Analyses were carried out with lmer package in R (release 3.0.1), SAS® (version 9.2; SAS Institute Inc, Cary, NC) and Matlab® (release 2010b; Mathworks Inc.).

## Results

### Characteristics of SB implementation

Fifteen patients implemented SB 6 days after being introduced to SB (interquartile: 1.5–15.5). Ten patients implemented SB with the aim of entirely suppressing binges, two with the aim of reducing binge frequency or binge food intake, one with these two aims and two did not provide information. Time between admission and SB presentation was not associated with binge frequency at admission (r = -0.08; p = 0.78). Binge frequency at SB presentation tended to be inversely associated with the delay between SB presentation and its first implementation (r = -0.48, p = 0.07), while reduction of binge frequency between admission and SB presentation was not (r = 0.18, p = 0.53). Overall, participants implemented SB twice (median number = 2, interquartile: 2–5) four days apart (median: 4, interquartile: 2–7) over the 38 days following first SB.

The number of binges the day before the first SB was similar to the number of binges before each of the following SBs (1.11 versus 1.45 binge, p = 0.15, model 1).

### Immediate effects of SB

During SB, patients ingested substantially less food compared to what they had originally planned (mean relative reduction (SD): 44% (20%)). The binge refractory period was substantially longer after an SB than after a regular binge (median (interquartile): 48 (6–168) vs 4 (1.5–24) hours respectively, p = 0.002). The relative reduction in food intake was associated with the relative binge refractory period (r = 0.59, p = 0.03): the less patients consumed food during a binge, the longer the refractory period.

Every SB day was associated with an average of 26% reduction in daily binge number the day after (p = 0.004, model 2). The daily binge number increased by 10% (mean) the day after each day without SB during the pre-SB period (p = 0.05, model 2) and by 66% during the post-SB period (p<0.0001, model 2). These two percentages were significantly different (+10% versus +66%, p<0.0001, model 2). Day-to-day variation in daily binge number was significantly different between SB days and no-SB days in the pre-SB period (-26% versus +10%, p = 0.0006, model 2). Similar results were found with the three other methods of imputations (variation in binge number: -23%, -14% and -26% for SB days during the post-SB period; +15%, +17% and +6% for no-SB days during the pre-SB period; +72%, +58% and +63% for no-SB days during the post-SB period; imputations A, B and C respectively, model 2).

**Fig 3:**
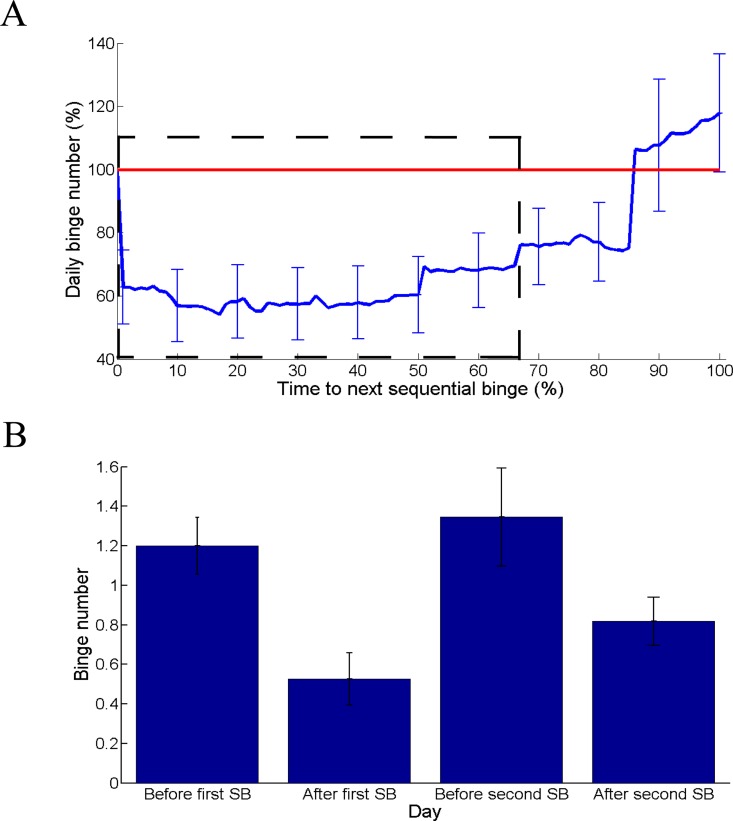
(A) Daily binge number as a function of the time to next sequential binge (SB). Daily binge number was computed relatively to the daily binge number of the previous SB day (in percent). For example, a daily binge number of 100% equals to the binge number of the previous SB day. Time between two consecutive SBs (e.g. SB A and SB B) was transformed into percentages so that a time interval of 0% corresponds to SB A day and a time interval of 100% to SB B day. All percentages of daily binge number within the dashed rectangle (time 1% to time 66%) are significantly lower than 100% (i.e. than the daily binge number on the previous SB day). Note that we used a threshold of p = 0.05 corrected for multiple comparisons with Benjamini false discovery rate. Eighty six SBs implemented over the follow-up period were included. (B) Number of binges the days before and after first and second SB. Mean and standard errors of the mean are reported.

Coherently, during the first two thirds of the time between two consecutive SBs, the binge number of a day was significantly lower than the one of the previous SB day (Fig 3A, and Fig 3B illustrating the variations in daily binge number between the day before and the day after SB for the first two SBs).

We then tested whether the observed effect of SB on the daily binge number was entirely or not entirely mediated by the reduction in binge food intake. Binge number on days after SB was 19% (p = 0.03, model 3) lower than the day before SB. This 19% reduction was not mediated by the reduction of binge food intake during SB. By contrast, there was a 68% increase in binge number the day after no-SB days. This percentage was significantly different from the 19% reduction the day after SB days (p<0.0001, model 3). Results were consistent with the three other methods of imputations (-16%, -7%, -18% for SB days vs. +89%, +69%, +60% for no-SB days for imputations A, B and C respectively).

### Long term effects of the sequential binge

A clinically significant depressive episode occurred in two patients after the first SB. It disappeared with the reduction in binge frequency and subsequent SB use without any change in medication. Patients attributed this depressive episode to an absence of binge craving to relieve their negative emotions during the binge refractory period.

Seven patients (47%) were free of binges for four consecutive weeks 16 weeks after the first SB (interquartile: 8–30) and thirteen patients (87%) experienced less than 2 binges per week for four consecutive weeks 13 weeks after the first SB.

### Experience of the sequential binge

Ten patients spontaneously reported their expectancy regarding SB. Eight of them reported that they had expected neither such a large reduction in food intake nor the long binge refractory period. The two remaining ones reported no surprise about the effects they observed after the first SB.

Failure in implementing SB at each binge was due to the urge to binge (5 ANB and 3 BN patients), a more intense pleasure provided by regular binges compared to SBs (3 ANB and 4 BN patients) or to the organizational and/or mental constraints related to the rules of ingestion (3 BN patients).

## Discussion

This article describes the sequential binge, a new therapeutic technique designed to actively target binge eating episodes. SB breaks up the binge into a repeated identical sequence of ingestion interspersed by incremental pauses. During the binge, this pattern of ingestion aims at promoting boredom toward the ingested foods and at turning cognitive control skills away from binge food restriction. With its design, SB can be implemented only if the patient intends to binge with a large amount of foods. However, SB was hypothesized to impact subsequent subjective binges. In a pilot group of binging patients (ANB, BN and BED) who did not respond to an intensive course of CBT complemented with SSRI, we observed: an acceptance rate (79%) close to the one of enhanced CBT (77%) [44]; food intake during SB almost halved compared to what patients had planned to eat; the refractory period was substantially longer after SBs compared to regular binges; daily binge number reduced substantially after each SB implementation while days without SB were associated with a proportional increase of binges the day after.

The substantial amount of uneaten food at the end of SB (patients ate only 44% of what they had planned) is likely higher than at the end of a regular binge. Indeed, others have found that most people eat entirely what they have planned [39,40].

The reduction in food intake during SB is likely due to the monotonous repetition of the sequence of food ingestion, as demonstrated in healthy participants [8]. The process deamed to mediate this effect is called specific satiety [8], the food specific signal triggering the end of ingestion of a food despite a sustained hunger [9,46]. Additional processes might also be involved: the visual perception of packages [47], a general boredom with the packages [48] and/or a reduction in the reward associated with the intake of large amount of highly palatable foods [49]. On the other hand, the reduction in food intake is unlikely to result from a reduced eating speed or a distraction effect coming from the reproduction of the ingestion sequence because these factors have not been shown to contribute to binge or regular food intake [50,51]. Neither is the reduction of food intake likely to be driven by a higher awareness of food ingestion during SB because cognitive resources are already recruited for reproducing the sequence of ingestion and the duration of pauses is limited.

The reduction of food intake during SBs partially accounted for the longer refractory period after SBs compared to regular binges. This could be influenced by a lower overall feeling of loss of control at the end of the binge due to the reduction of binge food intake [1]. This lower perception of loss of control would limit the strengthening of the diet after SB. In addition, some of the BN participants reported that they failed to implement SB because of the reduced attractiveness of SB. Thus, one can also stipulate that the lower food intake during SB may have reduced the reward associated with the intake of highly palatable foods [49] usually ingested during binges [6,7], and the desire to engage in a new binge accordingly.

However, this weak association between the reduction of food intake and the longer refractory period leaves room for another mechanism to account for the lengthening of the refractory period after SB. Likewise, food intake did not account for the reduction in binge number the day after SB. Instead, we postulate that the reduction of daily binge number after SB is related to the specific rules of ingestion during SB. Those rules are designed to train and reorient cognitive control abilities away from optimizing binge food restriction during the binge. Indeed, there is an increasing amount of evidence showing that patients plan food restriction and reinforce their dietary rules during binges, which in turn promotes subsequent binge episodes [1,10]. This break-up in the reinforcing mechanism of binge occurrence would favor a longer binge refractory period and reduce the daily binge number.

The higher increase in daily binge number on days without SB in the post-SB vs. pre-SB period could be interpreted as a side effect of SB. However, the increase in daily binge number after SBs occurred only in the second half of the time interval elapsed between two consecutive SBs, despite the presence of regular binges over the first half (Fig 3). The accumulation of regular binges between two consecutive SBs would have reinforced their occurrence [1] and overcome the reduction in binge number consecutive to SB. Therefore, we believe that this higher increase in daily binge numbers on non SB days in the post SB period results from the accumulation of regular binges occurring between two consecutive SBs rather than a side effect of SB. This points out the importance of warning patients that they should be implementing SB as often as possible to benefit fully from it.

Each SB was implemented to its end and patients never moved to a regular way of binging in the middle of SB, which supports its feasibility as an intervention for binge eating. However, we observed that SB was overall implemented less than regular binges. This could signal a motivational issue rather than difficulties in implementing SB. Indeed, patients preferred regular binges rather than SB because of a higher pleasure with regular binges. Motivation to implement SB could be raised by investigating patients' thoughts and feelings (and especially their sensation of loss of control) after they experienced SBs vs. regular binges. Understanding why patients implement the next SB only when they experience again the same number of binges as before the previous SB (Fig 3) might improve motivation to implement SB.

Our pilot study has several limitations. First no control group was included. Despite its substantial length, the unsuccessful implementation of binge management techniques, and the ABAB-like design with repeated SB implementations, the period between admission and SB presentation cannot be viewed as a valid control.

Second, one cannot completely exclude that the reduction of binge occurrence and extended refractory periods after SB was due to patients’ stronger motivation to reduce binge number at the time of SB or to an exceptionally high positive expectations towards SB. However, patients’ expectancy effects are unlikely because SB is per se a binge, hence goes against patients’ desire to avoid binges [1]. Additionally, patients did not expect, neither focused their efforts on, a reduction of daily binge number.

Third, the immediate effects of SB on binge number and on binge food intake are rather related to SB than to the other therapeutic interventions (CBT-E, SSRIs, group program) because SB positively impacts daily binge number the day after each of its implementation; and conversely, not doing SB negatively impacts daily binge number. Also note that the other therapeutic interventions were implemented without much success on daily binge number during the entire baseline period.

Fourth, our sample was small, highly selected (poor responders to CBT and SSRIs from in- or day patient settings), and heterogeneous regarding both eating disorders diagnoses and co-morbidities. Thus, one needs to be cautious about the generalization of our results on the possible benefits of SB. Moreover, assessing the clinical efficacy of SB was beyond the scope of this study that aimed to assess the feasibility and immediate effects of SB.

Fifth, as assessment of daily binge number and the amount of binge food ingested during SBs was based on patients' report, it might be affected by a recall bias and selective feedback. However binging patients report more accurately episodes of overeating compared to controls [52]. Moreover, if present, this bias should have equally affected the report of both SBs and regular binges. Overall, this data collection bias should be limited and only marginally affect the results.

## Conclusions and Perspectives

Despite their limitations, our results provide a proof of concept of SB and demonstrate that SB could be a feasible and therapeutic intervention for those patients who have failed to respond to an intensive course of CBT+SSRI. Of particular importance are the immediate effects of SB over food intake, daily binge number and binge refractory period. In addition, half of the participants reached binge abstinence for at least one month. This justifies the need of a randomized controlled trial to provide a full overview of the immediate and long term effects of SB. Especially, to improve the potential efficiency of SB, future studies might look for a cumulative effect when SB is re-implemented before the effect of the previous one has faded away.
